# Is inversion time dependent on relaxivity?

**DOI:** 10.1186/1532-429X-11-S1-T5

**Published:** 2009-01-28

**Authors:** June A Yamrozik, Mark Doyle, Vikas K Rathi, Diane A Vido, Ronald B Williams, Geetha Rayarao, Robert WW Biederman

**Affiliations:** Allegheny General Hospital, The Gerald McGinnis Cardiovascular Institute, Pittsburgh, PA USA

**Keywords:** Inversion Time, Gadopentetate Dimeglumine, Gadobenate Dimeglumine, High Relaxivity, Gadolinium Dosage

## Introduction

It is known that utilizing the proper inversion time (TI) is essential when diagnosing myocardial infarction. Many factors play a role in acquiring optimal nulling of the myocardium. Recently, higher relaxivity contrast agents have been advocated for use in infarct imaging. Specifically, MultiHance^®^ (gadobenate dimeglumine) has higher relaxivities (r_1_ and r_2_, 9.7^1^ and 12.5^1^) compared to standard gadolinium (gadopentetate dimeglumine) (r_1_ and r_2,_ 4.9^1^ and 6.3^1^). Will this higher relaxivity allow a lower dosage of contrast to be used to maintain a comparable TI null time compared to the conventional gadolinium dosage?

## Hypothesis

We hypothesize that a 0.15 mmol/kg dosage of MultiHance with its higher relaxivity will produce a lower TI null time in infarct patients compared to patients imaged with a standard dosage of 0.2 mmol/kg of gadolinium at 10 and 20 minutes post contrast.

## Methods

A total of 52 patients with GFR > 60 mL/min/1.73 m^2^ were imaged**.** Twenty-six (26) patients (19 M, 6 F), age 42–81 years, post-myocardial infarction underwent a standard cardiac MRI (CMR) utilizing a total of 0.2 mmol/kg gadolinium dosage (Magnevist-Berlex, New Jersey, USA). This was administered as a 0.5 mmol/kg for perfusion imaging followed by an additional 0.15 mmol/kg for viability (DHE) imaging. Twenty-six (26) patients (21 M, 5 F), age 43–83 years, post-myocardial infarction underwent a standard cardiac MRI (CMR) utilizing a total of 0.15 mmol/kg MultiHance dosage (Bracco Diagnostics, Princeton, N J, USA). This was administered as a 0.5 mmol/kg dosage for perfusion imaging followed by an additional 0.10 mmol/kg for viability imaging. The scans were acquired on a GE CV/i Excite Version 12, 1.5 T system (GE, Milwaukee, WI). The sequence utilized for optimum myocardial nulling was a standard 2D Gradient Echo IRP (FGR with inversion recovery prep). Manual selection of TI was performed independent of contrast media. An 8-channel or 4-channel cardiac coil was used, again independent of the contrast agent. The sequence parameters were as follows: TE: min, FA: 20, NEX: 2 trigger delay: adjusted to onset of diastole, 1RR interval and TI adjusted to null the myocardium. This sequence was performed at 10 and 20 minutes post-contrast administration.

## Results

All the patients successfully completed the CMR examination without difficulty, or impairment in infarct detection. Particular attention was focused on the contrast medium utilized, dosage and its effects on the inversion time (TI) at 10 and 20 minute post-contrast. At 10 minutes post-contrast, the inversion time with Magnevist was 158.7 ± 17.2 ms, which was not different from that obtained with MultiHance (154.3 ± 13.6 ms, p = 0.3). At 20 minutes post-contrast, the inversion time with Magnevist was 200.4 ± 28.8 ms, which again was not different from that obtained with MultiHance (193.0 ± 15.6 ms, p = 0.3).

## Conclusion

While the relaxivity of MultiHance compared to standard gadolinium is almost a factor of two higher, similar contrast null times are achieved by using a 75% dosage of MultiHance as compared to gadolinium. Relaxivity alone does not predict arithmetic decreases in TI times. Thus, while this is a higher dose than expected from relaxivity considerations alone, MultiHance administration is nevertheless potentially beneficial, especially in those situations when additional imaging might be necessary, since an additional 25% of contrast is held in reserve for further administration or saved, further minimizing any potential toxicity issues.

